# (*E*)-*N*′-(2-Bromo­benzyl­idene)-2-fluoro­benzohydrazide

**DOI:** 10.1107/S1600536811009937

**Published:** 2011-03-23

**Authors:** Dong-Fang Zhang, Da-Yong Liu, Chuan-Xun Li, Shan-Shan Huang, Bao-Jing Zhang

**Affiliations:** a College of Pharmaceutical Science, China Medical University, Shenyang 110001, People’s Republic of China; b Department of Chemistry and Chemical Engineering, Huanghuai University, Henan 463000, People’s Republic of China; c School of Pharmacy, Dalian Medical University, Dalian 116044, People’s Republic of China

## Abstract

The title compound, C_14_H_10_BrFN_2_O, adopts an *E* geometry about the C=N bond. The dihedral angle between the mean planes of the two benzene rings is 81.5 (6)°. In the crystal, mol­ecules are linked through inter­molecular N—H⋯O hydrogen bonds, forming chains running along the *b* axis.

## Related literature

For general background to the biological activity of Schiff bases, see: Bernardino *et al.* (2006 ▶); Ganjali *et al.* (2006 ▶). For related structures, see: Jiang (2006 ▶); Wardell *et al.* (2007 ▶); Zhu & He (2008 ▶); Li *et al.* (2009 ▶). For standard bond lengths, see: Allen *et al.* (1987 ▶).
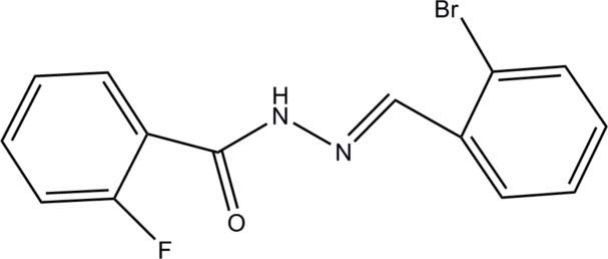


## Experimental

### 

#### Crystal data

C_14_H_10_BrFN_2_O
*M* *_r_* = 321.14
Orthorhombic, 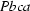

*a* = 11.853 (2) Å
*b* = 9.6507 (18) Å
*c* = 22.921 (4) Å
*V* = 2621.9 (8) Å^3^
*Z* = 8
Mo *K*α radiation
μ = 3.14 mm^−1^
*T* = 295 K
0.44 × 0.12 × 0.07 mm

#### Data collection

Bruker SMART CCD area-detector diffractometer
Absorption correction: multi-scan (*SADABS*; Sheldrick, 2003 ▶) *T*_min_ = 0.633, *T*_max_ = 0.798
15028 measured reflections
3111 independent reflections
1892 reflections with *I* > 2σ(*I*)
*R*_int_ = 0.064

#### Refinement

*R*[*F* ^2^ > 2σ(*F* ^2^)] = 0.043
*wR*(*F* ^2^) = 0.096
*S* = 1.00
2094 reflections
172 parameters
H-atom parameters constrained
Δρ_max_ = 0.31 e Å^−3^
Δρ_min_ = −0.57 e Å^−3^

### 

Data collection: *SMART* (Bruker, 2001 ▶); cell refinement: *SAINT-Plus* (Bruker, 2003 ▶); data reduction: *SAINT-Plus*; program(s) used to solve structure: *SHELXTL* (Sheldrick, 2008 ▶); program(s) used to refine structure: *SHELXTL*; molecular graphics: *SHELXTL*; software used to prepare material for publication: *SHELXTL*.

## Supplementary Material

Crystal structure: contains datablocks I, global. DOI: 10.1107/S1600536811009937/zq2093sup1.cif

Structure factors: contains datablocks I. DOI: 10.1107/S1600536811009937/zq2093Isup2.hkl

Additional supplementary materials:  crystallographic information; 3D view; checkCIF report

## Figures and Tables

**Table 1:** Hydrogen-bond geometry (Å, °)

*D*—H⋯*A*	*D*—H	H⋯*A*	*D*⋯*A*	*D*—H⋯*A*
N1—H1*A*⋯O1^i^	0.80	2.04	2.827 (3)	167

Symmetry code: (i) 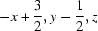.

**Fig. 1.**
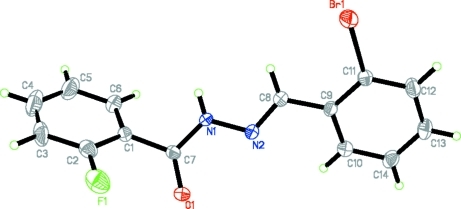
The molecular structure of the title compound (thermal ellipsoids are shown at the 30% probability level).

**Fig. 2.**
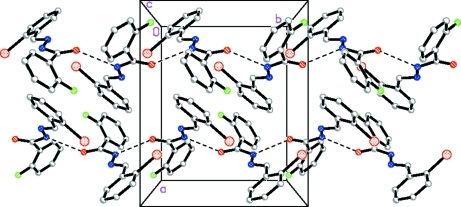
The crystal packing of the title compound viewed down the b axis. The dashed lines represent the hydrogen bonding interactions. Hydrogen atoms have been omitted for clarity.

## supplementary crystallographic information

### Comment

Schiff bases have attracted much attention due to their diverse range of bioactivities in pharmaceutical and agrochemical field (*e.g.* Bernardino *et al.*, 2006; Ganjali *et al.*, 2006). We now report the synthesis and crystal structure of the title compound (Fig. 1).

In the title compound, the Schiff base molecule adopts an *E* geometry with respect to the C=N bond, as shown in Fig. 1.The bond lengths and bond angles for (I) are within normal ranges (Allen *et al.*, 1987). The dihedral angle between the mean planes of the two benzene rings is 98.5 (6)°. The Schiff base moieties through intermolecular N—H···O hydrogen bonds form chains along the *b* axis, which helps to consolidate the crystal packing (Fig 2).

### Experimental

2-Fluorobenzohydrazide (0.1 mmol,15.4 mg) and 2-bromobenzaldehyde (0.1 mmol, 18.4 mg) were dissolved in a methanol solution (10 ml). The mixture was stirred at room temperature for 1 h and filtered. After keeping the filtrate in air for three days, colorless block-like crystals were formed.

### Refinement

The H1A atom bonded to N1 was located in a difference map and refined freely, other H atoms were placed in geometrically idealized positions and allowed to ride on their parent atoms, with *C*—H = 0.93 for phenyl, 0.97 Å for methylene H atoms, and with *U*_iso_(H) = 1.2*U*_eq_(C).

### Crystal data

**Table:**

C_14_H_10_BrFN_2_O	*F*(000) = 1280
*M**_r_* = 321.14	*D*_x_ = 1.627 Mg m^−^^3^
Orthorhombic, *P**b**c**a*	Mo *K*α radiation, λ = 0.71073 Å
Hall symbol: -P 2ac 2ab	Cell parameters from 2094 reflections
*a* = 11.853 (2) Å	θ = 2.5–21.5°
*b* = 9.6507 (18) Å	µ = 3.14 mm^−^^1^
*c* = 22.921 (4) Å	*T* = 295 K
*V* = 2621.9 (8) Å^3^	Block, yellow
*Z* = 8	0.44 × 0.12 × 0.07 mm

### Data collection

**Table:**

Bruker SMART CCD area-detector diffractometer	3111 independent reflections
Radiation source: fine-focus sealed tube	1892 reflections with *I* > 2σ(*I*)
graphite	*R*_int_ = 0.064
φ and ω scans	θ_max_ = 27.8°, θ_min_ = 2.5°
Absorption correction: multi-scan (*SADABS*; Sheldrick, 2003)	*h* = −15→15
*T*_min_ = 0.633, *T*_max_ = 0.798	*k* = −12→12
15028 measured reflections	*l* = −30→23

### Refinement

**Table:**

Refinement on *F*^2^	Primary atom site location: structure-invariant direct methods
Least-squares matrix: full	Secondary atom site location: difference Fourier map
*R*[*F*^2^ > 2σ(*F*^2^)] = 0.043	Hydrogen site location: inferred from neighbouring sites
*wR*(*F*^2^) = 0.096	H-atom parameters constrained
*S* = 1.00	*w* = 1/[σ^2^(*F*_o_^2^) + (0.0385*P*)^2^ + 0.3692*P*] where *P* = (*F*_o_^2^ + 2*F*_c_^2^)/3
2094 reflections	(Δ/σ)_max_ = 0.001
172 parameters	Δρ_max_ = 0.31 e Å^−^^3^
0 restraints	Δρ_min_ = −0.57 e Å^−^^3^

### Special details

**Table:**

Geometry. All e.s.d.'s (except the e.s.d. in the dihedral angle between two l.s. planes) are estimated using the full covariance matrix. The cell e.s.d.'s are taken into account individually in the estimation of e.s.d.'s in distances, angles and torsion angles; correlations between e.s.d.'s in cell parameters are only used when they are defined by crystal symmetry. An approximate (isotropic) treatment of cell e.s.d.'s is used for estimating e.s.d.'s involving l.s. planes.
Refinement. Refinement of *F*^2^ against ALL reflections. The weighted *R*-factor *wR* and goodness of fit *S* are based on *F*^2^, conventional *R*-factors *R* are based on *F*, with *F* set to zero for negative *F*^2^. The threshold expression of *F*^2^ > σ(*F*^2^) is used only for calculating *R*-factors(gt) *etc*. and is not relevant to the choice of reflections for refinement. *R*-factors based on *F*^2^ are statistically about twice as large as those based on *F*, and *R*- factors based on ALL data will be even larger.

### Fractional atomic coordinates and isotropic or equivalent isotropic displacement parameters (Å^2^)

**Table:**

	*x*	*y*	*z*	*U*_iso_*/*U*_eq_	
Br1	0.71246 (3)	−0.03729 (4)	1.026973 (15)	0.05866 (15)	
F1	0.93107 (15)	0.5216 (2)	0.78445 (10)	0.0708 (6)	
O1	0.71146 (17)	0.5201 (2)	0.83914 (9)	0.0438 (5)	
N1	0.69850 (17)	0.2891 (2)	0.85414 (10)	0.0328 (5)	
H1A	0.7221	0.2150	0.8442	0.039*	
N2	0.64592 (18)	0.3061 (2)	0.90719 (9)	0.0328 (5)	
C1	0.7828 (2)	0.3676 (3)	0.76568 (12)	0.0348 (7)	
C2	0.8798 (3)	0.4319 (3)	0.74739 (15)	0.0463 (8)	
C3	0.9276 (3)	0.4075 (4)	0.69309 (17)	0.0674 (11)	
H3	0.9928	0.4534	0.6815	0.081*	
C4	0.8758 (4)	0.3137 (5)	0.65694 (17)	0.0805 (13)	
H4	0.9065	0.2961	0.6203	0.097*	
C5	0.7808 (4)	0.2461 (4)	0.67351 (16)	0.0708 (11)	
H5	0.7473	0.1818	0.6487	0.085*	
C6	0.7341 (3)	0.2737 (3)	0.72758 (13)	0.0503 (9)	
H6	0.6683	0.2281	0.7386	0.060*	
C7	0.7291 (2)	0.4011 (3)	0.82290 (12)	0.0313 (6)	
C8	0.6410 (2)	0.1979 (3)	0.93891 (12)	0.0343 (7)	
H8	0.6714	0.1148	0.9257	0.041*	
C9	0.5869 (2)	0.2055 (3)	0.99638 (12)	0.0323 (6)	
C10	0.5124 (2)	0.3127 (3)	1.00943 (12)	0.0396 (7)	
H10	0.4954	0.3779	0.9809	0.047*	
C11	0.6079 (2)	0.1080 (3)	1.03970 (12)	0.0365 (7)	
C12	0.5580 (3)	0.1179 (4)	1.09390 (13)	0.0493 (8)	
H12	0.5728	0.0514	1.1223	0.059*	
C13	0.4864 (2)	0.2259 (4)	1.10586 (14)	0.0503 (9)	
H13	0.4533	0.2329	1.1425	0.060*	
C14	0.4631 (2)	0.3244 (3)	1.06384 (13)	0.0459 (8)	
H14	0.4147	0.3978	1.0721	0.055*	

### Atomic displacement parameters (Å^2^)

**Table:**

	*U*^11^	*U*^22^	*U*^33^	*U*^12^	*U*^13^	*U*^23^
Br1	0.0711 (3)	0.0520 (2)	0.0528 (2)	0.02188 (19)	0.01343 (18)	0.01330 (17)
F1	0.0498 (11)	0.0695 (14)	0.0930 (16)	−0.0141 (10)	0.0000 (11)	0.0123 (13)
O1	0.0615 (14)	0.0263 (12)	0.0437 (12)	−0.0043 (10)	0.0116 (10)	−0.0023 (10)
N1	0.0453 (13)	0.0249 (13)	0.0281 (13)	0.0038 (10)	0.0084 (11)	−0.0023 (10)
N2	0.0380 (13)	0.0312 (14)	0.0292 (13)	−0.0003 (11)	0.0066 (11)	−0.0022 (11)
C1	0.0383 (16)	0.0328 (16)	0.0334 (16)	0.0023 (14)	0.0075 (14)	0.0047 (13)
C2	0.0417 (18)	0.043 (2)	0.054 (2)	0.0023 (15)	0.0002 (16)	0.0109 (16)
C3	0.047 (2)	0.085 (3)	0.070 (3)	0.014 (2)	0.029 (2)	0.029 (2)
C4	0.087 (3)	0.111 (4)	0.044 (2)	0.032 (3)	0.022 (2)	0.006 (2)
C5	0.087 (3)	0.083 (3)	0.043 (2)	0.013 (2)	0.012 (2)	−0.012 (2)
C6	0.061 (2)	0.050 (2)	0.040 (2)	0.0027 (16)	0.0093 (16)	−0.0049 (16)
C7	0.0342 (15)	0.0264 (16)	0.0332 (16)	−0.0022 (12)	0.0005 (13)	0.0016 (13)
C8	0.0373 (16)	0.0333 (17)	0.0324 (16)	0.0012 (14)	0.0028 (13)	−0.0018 (13)
C9	0.0356 (15)	0.0326 (17)	0.0288 (15)	−0.0048 (13)	0.0056 (13)	−0.0037 (13)
C10	0.0430 (17)	0.0382 (18)	0.0375 (18)	0.0015 (15)	0.0023 (14)	0.0005 (14)
C11	0.0377 (16)	0.0353 (17)	0.0364 (17)	−0.0014 (13)	0.0061 (13)	0.0010 (13)
C12	0.054 (2)	0.056 (2)	0.0378 (19)	−0.0018 (17)	0.0098 (16)	0.0104 (16)
C13	0.0483 (19)	0.065 (2)	0.0377 (19)	−0.0014 (18)	0.0157 (16)	−0.0007 (17)
C14	0.0407 (17)	0.051 (2)	0.046 (2)	0.0024 (16)	0.0131 (15)	−0.0069 (16)

### Geometric parameters (Å, °)

**Table:**

Br1—C11	1.894 (3)	C5—C6	1.383 (4)
F1—C2	1.356 (4)	C5—H5	0.9300
O1—C7	1.226 (3)	C6—H6	0.9300
N1—C7	1.346 (3)	C8—C9	1.467 (4)
N1—N2	1.376 (3)	C8—H8	0.9300
N1—H1A	0.8011	C9—C11	1.390 (4)
N2—C8	1.274 (3)	C9—C10	1.393 (4)
C1—C2	1.372 (4)	C10—C14	1.382 (4)
C1—C6	1.385 (4)	C10—H10	0.9300
C1—C7	1.494 (4)	C11—C12	1.379 (4)
C2—C3	1.387 (5)	C12—C13	1.372 (4)
C3—C4	1.372 (5)	C12—H12	0.9300
C3—H3	0.9300	C13—C14	1.381 (4)
C4—C5	1.355 (6)	C13—H13	0.9300
C4—H4	0.9300	C14—H14	0.9300
			
C7—N1—N2	119.7 (2)	O1—C7—C1	122.8 (3)
C7—N1—H1A	118.1	N1—C7—C1	114.1 (2)
N2—N1—H1A	121.1	N2—C8—C9	119.4 (3)
C8—N2—N1	115.3 (2)	N2—C8—H8	120.3
C2—C1—C6	116.9 (3)	C9—C8—H8	120.3
C2—C1—C7	121.9 (3)	C11—C9—C10	117.6 (3)
C6—C1—C7	121.1 (3)	C11—C9—C8	122.0 (3)
F1—C2—C1	118.2 (3)	C10—C9—C8	120.5 (3)
F1—C2—C3	119.2 (3)	C14—C10—C9	121.5 (3)
C1—C2—C3	122.6 (3)	C14—C10—H10	119.2
C4—C3—C2	118.1 (3)	C9—C10—H10	119.2
C4—C3—H3	121.0	C12—C11—C9	121.3 (3)
C2—C3—H3	121.0	C12—C11—Br1	118.1 (2)
C5—C4—C3	121.3 (4)	C9—C11—Br1	120.5 (2)
C5—C4—H4	119.3	C13—C12—C11	119.8 (3)
C3—C4—H4	119.3	C13—C12—H12	120.1
C4—C5—C6	119.4 (4)	C11—C12—H12	120.1
C4—C5—H5	120.3	C12—C13—C14	120.5 (3)
C6—C5—H5	120.3	C12—C13—H13	119.8
C5—C6—C1	121.6 (3)	C14—C13—H13	119.8
C5—C6—H6	119.2	C13—C14—C10	119.2 (3)
C1—C6—H6	119.2	C13—C14—H14	120.4
O1—C7—N1	123.0 (2)	C10—C14—H14	120.4

### Hydrogen-bond geometry (Å, °)

**Table:**

*D*—H···*A*	*D*—H	H···*A*	*D*···*A*	*D*—H···*A*
N1—H1A···O1^i^	0.80	2.04	2.827 (3)	167

Symmetry codes: (i) −*x*+3/2, *y*−1/2, *z*.
